# Seventy‐Two–Hour Emergency Department Revisits in the Pediatric Population at King Abdullah Specialist Children′s Hospital, Riyadh, Saudi Arabia: A Morbidity Analysis

**DOI:** 10.1155/ijpe/1897862

**Published:** 2026-06-20

**Authors:** Mohammed AlAteeq, Shaden Althuwaini, Haya Alsubaie, Lulwah Al-alshaikh, Ghaid Alotaibi, Sarah Alghuraybi, Aamir Omair

**Affiliations:** ^1^ Ambulatory Care Services, Ministry of National Guard-Health Affairs, Riyadh, Saudi Arabia, ngha.med.sa; ^2^ King Abdullah International Medical Research Center, Riyadh, Saudi Arabia, kaimrc.med.sa; ^3^ College of Medicine, King Saud bin Abdulaziz University for Health Sciences, Riyadh, Saudi Arabia, ksau-hs.edu.sa

**Keywords:** children, ED revisit, emergency department, return visit, Saudi Arabia

## Abstract

**Background:**

Recent data suggest that pediatric emergency department (ED) revisits within 72 h have increased globally, particularly during recent respiratory illness surges, potentially straining healthcare resources. This study is aimed at identifying reasons for these revisits and describe management outcomes for children aged 0–14 years.

**Methods:**

A cross‐sectional chart review was conducted at a tertiary hospital in Riyadh from March 1, 2022, to April 30, 2023. The study included Saudi nationals aged 0–14 years who revisited the ED within 72 h of their initial visit; noncitizen children were excluded. Data captured included demographics, medical history, and details of both initial and revisits (reason for visit, assessment, and management).

**Results:**

Three hundred Saudi children were included; 31% were aged 0–3 years, and approximately 60% were male. Twenty‐six percent had chronic medical conditions, with asthma exacerbation being most common (12%). Persistent cough accounted for the highest proportion of revisits (34%). Antibiotics were prescribed in 28% of initial visits and 29% of revisits. Reassurance was provided in 27% of initial visits and 27% of revisits. The 0–3 year age group showed significantly higher rates of admission and discharge.

**Conclusion:**

Persistent respiratory symptoms (especially cough and dyspnea) were the most frequent reasons for pediatric ED revisits, with asthma as the prevalent chronic condition. Improving initial diagnosis and management is essential to reduce avoidable revisits. Future research comparing presurge and postsurge periods could clarify if revisit reasons change over time.

## 1. Introduction

A revisit (RV) to the pediatric emergency department (ED) is defined as an unscheduled presentation occurring shortly after discharge from an initial visit [1]. Globally, there has been an increase in pediatric ED RVs, which can place strain on healthcare resources and staff capacity [2–4]. These RVs stem from diverse medical conditions affecting respiratory, gastrointestinal, and genitourinary systems among others, often reflecting challenges related to initial diagnosis, management, or evolving clinical symptoms [1–6].

Many studies have investigated the characteristics and risk factors associated with pediatric ED RVs. A recent systematic review encompassing 28 studies across multiple countries identified factors such as younger age, high acuity at initial visit, and language barriers as stronger predictors for RVs compared with socioeconomic status or previous healthcare usage [7]. For example, a retrospective study in Turkey indicated younger children had higher rates of RVs within 24 h, underscoring the vulnerability of this age group to recurrent care needs [8].

During the COVID‐19 pandemic, variations in pediatric ED visits and return rates were observed globally. A South Korean hospital study revealed that RV rates peaked among infants under 1 year old with infectious and respiratory complaints prominently represented, pointing to temporal and epidemiological influences on RV patterns [9]. Similarly, research at a Swiss tertiary children′s hospital showed that infants and toddlers comprised most RVs, though the majority appeared unnecessary, highlighting opportunities to refine discharge planning [10].

Additionally, one study analyzed ED RVs across European nations to improve operational efficiency of pediatric EDs and found that gastrointestinal and respiratory symptoms were the most frequently reported issues leading to RVs [1]. Another research project evaluated RV rates and influencing factors at KK Women′s and Children′s Hospital in Singapore, identifying respiratory and gastrointestinal illnesses as the primary diagnoses, particularly affecting children aged 3 and under [2]. In a prospective study conducted in Rotterdam, researchers aimed to characterize the timing and nature of RVs in pediatric patients presenting with symptoms like dyspnea, fever, or vomiting/diarrhea. The study revealed that patients with vomiting/diarrhea often returned to the ED within 1 day, whereas those with fever or dyspnea typically revisited after 2 days [11].

Furthermore, a study focusing on pediatric patients with back pain during their initial ED visit utilized the United States Children′s Hospital database to assess the incidence of serious diagnoses during subsequent visits, finding that 0.3%–0.4% of participants were diagnosed with serious conditions within 30 days of their initial visit [12].

Locally, research from King Abdullah Specialist Children′s Hospital (KASCH) in Riyadh has begun to shed light on pediatric ED RVs within the Saudi Arabian context [6]. This work primarily focused on children with chronic illnesses, identifying asthma exacerbation and allergies as leading contributors to RVs within 72 h. Other regional studies echo the importance of respiratory conditions but often lack comprehensive age group coverage or focus narrowly on acute lower respiratory diseases [13].

Although international and some regional literature on pediatric ED RVs has grown substantially, institution‐specific data from tertiary pediatric centers in Saudi Arabia remain limited, particularly studies that encompass all age groups and presenting complaints rather than focusing solely on chronic disease subgroups. Western multicenter data have provided valuable insight into RV causes and timing; however, the generalizability of these findings to different healthcare settings is constrained by variation in healthcare infrastructure, insurance systems, cultural factors, and patterns of disease presentation.

This study is aimed at addressing this gap at the institutional level by comprehensively analyzing reasons for ED RVs and management outcomes among Saudi children aged 0–14 years at KASCH—a large tertiary pediatric center in Riyadh. By focusing on a broad pediatric population and including all causes of RV, this research seeks to provide institution‐specific insights that may inform patient care pathways and quality improvement initiatives at comparable tertiary facilities, with the understanding that broader regional applicability requires future multicenter investigation.

## 2. Methods

This study is a retrospective observational cross‐sectional chart review conducted at the ED of KASCH in Riyadh, Saudi Arabia, recognized as one of the largest pediatric emergency service departments in the region, equipped with advanced diagnostic and therapeutic facilities. The ED is augmented by tertiary care services across all subspecialties, with an approximate daily visit rate of 200. The study encompassed all Saudi pediatric patients aged 0–14 years who revisited the ED within 72 h of their initial visit for both acute and chronic conditions, during the 14‐month period from March 1, 2022, to April 30, 2023. The upper age limit for the pediatric age group at our institute is 14 years, which informed the selection of the study population′s age range. During this 14‐month study period, the total number of ED visits recorded was approximately 78,000. An international study indicates that the rate of ED RVs within 72 h ranges from 2.2%–3.5% of all ED visits [11]. The sample of 300 patients was calculated to be statistically representative of the estimated 2730 RVs based on accepted sample size determination methods. Using the Raosoft software with parameters including 95% CI, a margin of error of 2.08%, and an assumed RV rate of 3.5%, a minimum sample size of 300 was determined to sufficiently capture the population characteristics.

Following the acquisition of eligible patients′ medical record numbers from the electronic medical records database at KASCH, simple random sampling was employed to select study subjects using an online random number generator. Simple random sampling was applied to the entire pool of eligible RV encounters without stratification by month or season; consequently, the seasonal distribution observed in the 300‐case sample reflects the underlying seasonal distribution of all eligible RVs during the study period. The 14‐month study period (March 2022–April 2023) means that the months of March and April each appear twice in the calendar window. This is acknowledged as a potential source of seasonal imbalance and should be considered when interpreting seasonal trends; however, because random sampling was applied across the full eligible pool without monthly weighting, the sample proportions are expected to mirror the population proportions. A data collection sheet was utilized to compile information on demographics, medical history, and details of the initial and subsequent visits, including reasons for attendance, assessments, and management strategies. Patients′ ages were categorized into four groups: under 3 years, 3 to under 6 years, 6 to under 9 years, and 9 to 14 years. Patients presenting with RVs for reasons unrelated to their initial complaint were excluded to ensure the study focused exclusively on RVs representing a continuum of the original presenting condition.

## 3. Results

A total of 300 Saudi children were included in the study. The cohort was predominantly young, with 31% (*n* = 92) aged 0–3 years, 20% (*n* = 59) aged 3–6 years, 23% (*n* = 68) aged 6–9 years, and 27% (*n* = 81) aged 9–14 years. Approximately 60% (*n* = 181) were male. Twenty‐six percent (*n* = 79) had at least one documented chronic medical condition; of these, asthma was the most prevalent (12%, *n* = 37), followed by diabetes mellitus (DM) (2.3%, *n* = 7), scoliosis (1%, *n* = 3), and autism spectrum disorder (ASD) (1%, *n* = 3). Fifteen percent (*n* = 44) were on chronic medications, and 4% (*n* = 12) had a history of recent admission. Full demographic and medical characteristics are summarized in Table 1.

**Table 1:** Demographic and medical characteristics of participants (*N* = 300).

Characteristic	*n* (%)
**Age**	
0–3 years	92 (30.6%)
3–6 years	59 (19.6%)
6–9 years	68 (22.6%)
9–14 years	81 (27%)

**Gender**	
Female	119 (40%)
Male	181 (60%)

**Chronic medical conditions**	
Asthma	37 (12%)
Scoliosis	3 (1%)
DM	7 (2.3%)
ASD	3 (1%)
Others	41 (14%)
No	221 (74%)

**History of surgical conditions**	
No	287 (95.6%)
Yes	13 (4.3%)

**History of chronic medication**	
No	256 (85%)
Yes	44 (15%)

**Recent admission**	
No	288 (96%)
Yes	12 (4%)

Among participants, the most frequently reported reasons for ED RVs were persistent cough (34%, *n* = 102), persistent fever (25%, *n* = 76), vomiting (20%, *n* = 61), dyspnea (9.7%, *n* = 29), and diarrhea (9%, *n* = 27) (Table 2).

**Table 2:** Reasons for ED revisit among participants (*N* = 300).

Characteristic	*n* (%)
**Persistent cough**	102 (34%)
**Vomiting**	61 (20%)
**Persistent fever**	76 (25%)
**Dyspnea**	29 (9.7%)
**Diarrhea**	27 (9%)
**Sore throat**	24 (8%)
**Redose**	22 (7.3%)
**Runny nose**	19 (6.3%)
**Abdominal pain**	14 (4.7%)
**Fatigue**	13 (4.3%)
**Others**	99 (33%)

Persistent cough was the leading RV complaint (34%), and this symptom was also the primary reason for the index ED visit in the majority of these cases, consistent with the overall pattern in which upper respiratory tract infection (URTI) was the most common initial diagnosis (52%). Similarly, persistent fever accounted for 25% of RVs and was concordant with the initial presentation in the majority of those encounters, often in the context of URTI or viral illness. This pattern suggests that a substantial proportion of RVs represent unresolved or slowly evolving illness rather than new clinical events.

Regarding medications prescribing, antibiotics were given in 28% (*n* = 84) of initial visits and 29% (*n* = 86) of RVs (Tables 3 and 4). Antibiotics prescription was the most frequently prescribed intervention at both time points. Among patients with an initial diagnosis of URTI—which accounted for 52% of all index diagnoses—a substantial proportion received antibiotics at the initial visit despite the predominantly viral etiology of URTI in children. The maintenance of a comparable antibiotic prescription rate at RV (29%) suggests limited de‐escalation between encounters. Among the RV diagnoses, URTI remained the most common assessment (40%), followed by gastroenteritis (GE) (12%), viral illness (6.7%), and pneumonia (6%). Whether antibiotic prescriptions at RV were continuations of index‐visit prescriptions or represented new prescriptions could not be fully disaggregated in this retrospective dataset; this is noted as a limitation. Nonetheless, the persistence of antibiotic prescribing across both encounters in the context of predominantly viral respiratory diagnoses raises concerns relevant to antimicrobial stewardship in the pediatric emergency setting.

**Table 3:** Initial management and outcomes among participants (*N* = 300).

Characteristic	
**Initial management** ^∗^	*n* (%)
Antibiotic prescription	84 (28%)
Reassurance	82 (27%)
X‐ray	55 (18%)
Blood test	47 (16%)
Normal saline	37 (12%)
Corticosteroid	30 (10%)
Nasal drops	31 (10%)
Antipyretic	27 (9%)
Urinalysis	24 (8%)
Culture	19 (6.3%)
Oral rehydration solution (ORS)	19 (6.3%)
Salbutamol	18 (6%)
Antiemetic	17 (5.7%)
Pain reliever	8 (2.7%)
Other management	122 (41%)
**Initial outcome**	
LWBS	1 (0.3%)
Admission	3 (1%)
DAMA	1 (0.3%)
Discharged	293 (98%)
Referral	2 (0.7%)
^∗^Multichoice questions	

*Note:* Asterisk means : Multichoice questions / Multipe answers

**Table 4:** Management and outcomes during revisit among participants.

Revisit management ^∗^	*n* (%)
Antibiotic prescription	86 (29%)
Reassurance	81 (27%)
Blood test	59 (20%)
Normal saline	57 (19%)
X‐ray	49 (16%)
Pain reliever	44 (15%)
Salbutamol	27 (9%)
Nebulization	27 (9%)
Antiemetic	23 (7.7%)
Corticosteroid	20 (6.7%)
Ipratropium bromide	15 (5%)
Nasal drops	14 (4.7%)
Other management	119 (40%)

**Revisit outcome**	
Admission	47 (16%)
Discharged	243 (81%)
Referral	9 (3%)
Upgrade to the resuscitation area	1 (0.3%)
^∗^Multichoice questions	

*Note:* Asterisk means : Multichoice questions / Multipe answers

The most prevalent initial management strategies employed were prescriptions (28%), reassurance (27%), imaging studies (18%), and blood tests (16%). Concerning initial outcomes, the majority (98%) were discharged, 1% were admitted, 0.3% discharged against medical advice (DAMA), 0.7% were referred, whereas a small percentage (0.3%) left without being seen (LWBS). Upon revisiting management, strategies employed were prescriptions (29%), imaging studies (16%), reassurance (27%), blood tests (20%), and other management (40%). Regarding RV outcomes, 16% were admitted, 81% were discharged, 3% were referred, and 0.3% were escalated to the resuscitation area (Tables 3 and 4).

The outcomes of pediatric RVs varied significantly by age (*p* < 0.001), with higher rates of discharge and admission observed in the 0–3 years group compared with others. Additionally, outcomes differed significantly by gender (*p* = 0.033), with elevated rates of discharge and admission noted among males (Table 5). Vomiting also showed significant differences by age (*p* = 0.005), with higher rates in the 3–6 years and 9–14 years groups compared with others. Dyspnea exhibited significant variation (*p* < 0.001), with increased rates in the younger age groups (0–3 years). Abdominal pain (*p* = 0.015) and persistent fever (*p* = 0.046) demonstrated significant differences by age, with higher rates observed in the 9–14 years group compared with others. Furthermore, the occurrence of other unspecified symptoms varied significantly among age groups (*p* = 0.028), with the 9–14 years group exhibiting the highest prevalence. Gender variation in the reasons for RV was evident, with males exhibiting higher rates of dyspnea, whereas females demonstrated a greater need for repeat dosing.

**Table 5:** Demographics versus outcome of revisits.

	Outcome of revisit				
Characteristic	Admission, *n* = 47	Discharged, *n* = 243	Referral, *n* = 9	Upgrade to resuscitation area, *n* = 1	*p* value^a^
**Age**					**< 0.001**
0–3 years	22 (47%)	69 (28%)	0 (0%)	1 (100%)	
3–6 years	11 (23%)	46 (19%)	2 (22%)	0 (0%)	
6–9 years	5 (11%)	63 (26%)	0 (0%)	0 (0%)	
9–14 years	9 (19%)	65 (27%)	7 (78%)	0 (0%)	

**Gender**					**0.033**
Female	11 (23%)	103 (42%)	5 (56%)	0 (0%)	
Male	36 (77%)	140 (58%)	4 (44%)	1 (100%)	

**Chronic medical conditions**					0.3
No	29 (62%)	181 (74%)	7 (78%)	1 (100%)	
Yes	18 (38%)	62 (26%)	2 (22%)	0 (0%)	

**Surgical conditions**					0.13
No	42 (89%)	235 (97%)	9 (100%)	1 (100%)	
Yes	5 (11%)	8 (3.3%)	0 (0%)	0 (0%)	

**Chronic medication**					0.3
No	36 (77%)	211 (87%)	8 (89%)	1 (100%)	
Yes	11 (23%)	32 (13%)	1 (11%)	0 (0%)	


*Note:* Bolded numbers in last column means statistical significance

^a^Fisher′s exact test.

The majority of patients (99%) initially presented via self‐referral, with 38% arriving in the evening, 27% in the morning, and 35% at night. Seasonally, visits were fairly evenly distributed across fall (28%), spring (25%), summer (25%), and winter (23%). In terms of symptom duration, most patients reported complaints lasting 1–4 days (79%), followed by 5–7 days (6.7%). Initial diagnoses were predominantly associated with URTIs (52%), gastrointestinal disorders (12%), asthma exacerbation (6.7%), and pneumonia (4%), among others. Other patient characteristics and healthcare utilization patterns at initial visit and RV are summarized in Table 6. Participants diagnosed with conditions other than those listed at the initial visit and RV were significantly associated with the outcome of the RV (Table 7). Among patients presenting with cough or fever at the initial visit, a notable proportion continued to report the same symptom at the time of RV: 84 patients (28%) had persistent cough at both visits, and 115 patients (38.3%) had persistent fever at both visits (Table 8).

**Table 6:** Participants characteristics and healthcare utilization patterns at initial visits and revisit (*N* = 300).

Characteristic	*n*(%)
**Initial source of patient**	
Physician‐initiated recall for follow‐up	2 (0.7%)
Referral	1 (0.3%)
Self‐referral	297 (99%)

**Initial time of day of visit**	
Evening	115 (38%)
Morning	80 (27%)
Night	105 (35%)

**Initial season of the year**	
Fall	84 (28%)
Spring	74 (25%)
Summer	74 (25%)
Winter	68 (23%)

**Initial duration of complaints**	
1–12 h	19 (6.3%)
1–4 day	238 (79%)
14–28 days	5 (1.7%)
2–15 min	13 (4.3%)
5–7 days	20 (6.7%)
Not mentioned	5 (1.7%)

**Initial diagnosis**	
URTI	157 (52%)
Urinary tract infection (UTI)	8 (2.7%)
Asthma exacerbation	20 (6.7%)
GE	35 (12%)
Pneumonia	12 (4%)
Viral illness	9 (3%)
Bronchiolitis	6 (2%)
Other	81 (27%)

**Source of patient at revisit**	
Physician‐initiated recall for follow‐up	40 (13%)
Self‐referral	260 (87%)

**Time of day at revisit**	
Evening	107 (36%)
Morning	106 (35%)
Night	87 (29%)

**Season of the year at revisit**	
Fall	85 (28%)
Spring	74 (25%)
Summer	73 (24%)
Winter	68 (23%)

**Duration of complaint at revisit**	
1–4 days	217 (72%)
16 days	1 (0.3%)
5–9 days	56 (19%)
5 min–few hours	24 (8%)
Not mentioned	2 (0.6%)

**Diagnosis/assessment at revisit**	
URTI	120 (40%)
GE	36 (12%)
Asthma exacerbation	15 (5%)
Bronchiolitis	4 (1.3%)
Pneumonia	18 (6%)
Viral illness	20 (6.7%)
UTI	10 (3.3%)
Constipation	7 (2.3%)

**Table 7:** Outcome and characteristics of revisits.

	Outcome of revisit				
Characteristic	Admission, *n* = 47	Discharged, *n* = 243	Referral, *n* = 9	Upgrade to resuscitation area, *n* = 1	*p* value
**Source of patient**					> 0.9
Physician‐initiated recall for follow‐up	0 (0%)	2 (0.8%)	0 (0%)	0 (0%)	
Referral	0 (0%)	1 (0.4%)	0 (0%)	0 (0%)	
Self‐referral	47 (100%)	240 (99%)	9 (100%)	1 (100%)	

**Time of day of visit**					0.5
Evening	17 (36%)	93 (38%)	5 (56%)	0 (0%)	
Morning	16 (34%)	61 (25%)	2 (22%)	1 (100%)	
Night	14 (30%)	89 (37%)	2 (22%)	0 (0%)	

**Season of the year**					0.8
Fall	11 (23%)	70 (29%)	3 (33%)	0 (0%)	
Spring	13 (28%)	59 (24%)	2 (22%)	0 (0%)	
Summer	9 (19%)	61 (25%)	3 (33%)	1 (100%)	
Winter	14 (30%)	53 (22%)	1 (11%)	0 (0%)	

**Duration of complains**					0.13
1–12 h	2 (4.3%)	14 (5.8%)	3 (33%)	0 (0%)	
1–4 day	39 (83%)	194 (80%)	4 (44%)	1 (100%)	
14–28 days	0 (0%)	5 (2.1%)	0 (0%)	0 (0%)	
2–15 min	2 (4.3%)	9 (3.7%)	2 (22%)	0 (0%)	
5–7 days	4 (8.5%)	16 (6.6%)	0 (0%)	0 (0%)	
Not mentioned	0 (0%)	5 (2.1%)	0 (0%)	0 (0%)	

**Initial diagnosis**					
**URTI**					0.2
No	24 (51%)	112 (46%)	7 (78%)	0 (0%)	
Yes	23 (49%)	131 (54%)	2 (22%)	1 (100%)	

**UTI**					> 0.9
No	46 (98%)	236 (97%)	9 (100%)	1 (100%)	
Yes	1 (2.1%)	7 (2.9%)	0 (0%)	0 (0%)	

**Asthma** exacerbation					0.9
No	45 (96%)	225 (93%)	9 (100%)	1 (100%)	
Yes	2 (4.3%)	18 (7.4%)	0 (0%)	0 (0%)	

**GE**					0.5
No	44 (94%)	212 (87%)	8 (89%)	1 (100%)	
Yes	3 (6.4%)	31 (13%)	1 (11%)	0 (0%)	

**Pneumonia**					> 0.9
No	45 (96%)	233 (96%)	9 (100%)	1 (100%)	
Yes	2 (4.3%)	10 (4.1%)	0 (0%)	0 (0%)	

**Viral illness**					0.11
No	43 (91%)	238 (98%)	9 (100%)	1 (100%)	
Yes	4 (8.5%)	5 (2.1%)	0 (0%)	0 (0%)	

**Bronchiolitis**					> 0.9
No	46 (98%)	238 (98%)	9 (100%)	1 (100%)	
Yes	1 (2.1%)	5 (2.1%)	0 (0%)	0 (0%)	

**Others**					**< 0.001**
No	38 (81%)	179 (74%)	1 (11%)	1 (100%)	
Yes	9 (19%)	64 (26%)	8 (89%)	0 (0%)	

**Source of patient at revisit**					0.054
Physician‐initiated recall for follow‐up	4 (8.5%)	32 (13%)	4 (44%)	0 (0%)	
Self‐referral	43 (91%)	211 (87%)	5 (56%)	1 (100%)	

**Time of day in revisit**					0.5
Evening	13 (28%)	89 (37%)	5 (56%)	0 (0%)	
Morning	20 (43%)	84 (35%)	2 (22%)	0 (0%)	
Night	14 (30%)	70 (29%)	2 (22%)	1 (100%)	

**Season of the year in revisit**					0.8
Fall	11 (23%)	71 (29%)	3 (33%)	0 (0%)	
Spring	13 (28%)	59 (24%)	2 (22%)	0 (0%)	
Summer	9 (19%)	60 (25%)	3 (33%)	1 (100%)	
Winter	14 (30%)	53 (22%)	1 (11%)	0 (0%)	

**Duration of complaint in revisit**					> 0.9
1–4 days	36 (77%)	172 (71%)	8 (89%)	1 (100%)	
16 days	0 (0%)	1 (0.4%)	0 (0%)	0 (0%)	
5–9 days	9 (19%)	46 (19%)	1 (11%)	0 (0%)	
5 minutes–few hours	2 (4.3%)	22 (9.1%)	0 (0%)	0 (0%)	
Not mentioned	0 (0%)	2 (0.8%)	0 (0%)	0 (0%)	

**Diagnosis at revisit**					
**URTI**					0.12
No	33 (70%)	140 (58%)	7 (78%)	0 (0%)	
Yes	14 (30%)	103 (42%)	2 (22%)	1 (100%)	
**GE**					> 0.9
No	42 (89%)	213 (88%)	8 (89%)	1 (100%)	
Yes	5 (11%)	30 (12%)	1 (11%)	0 (0%)	

**Asthma** exacerbation					0.7
No	46 (98%)	229 (94%)	9 (100%)	1 (100%)	
Yes	1 (2.1%)	14 (5.8%)	0 (0%)	0 (0%)	

**Bronchiolitis**					0.6
No	46 (98%)	240 (99%)	9 (100%)	1 (100%)	
Yes	1 (2.1%)	3 (1.2%)	0 (0%)	0 (0%)	

**Pneumonia**					0.7
No	43 (91%)	229 (94%)	9 (100%)	1 (100%)	
Yes	4 (8.5%)	14 (5.8%)	0 (0%)	0 (0%)	

**Viral illness**					0.3
No	41 (87%)	229 (94%)	9 (100%)	1 (100%)	
Yes	6 (13%)	14 (5.8%)	0 (0%)	0 (0%)	

**UTI**					> 0.9
No	46 (98%)	234 (96%)	9 (100%)	1 (100%)	
Yes	1 (2.1%)	9 (3.7%)	0 (0%)	0 (0%)	

**Constipation**					0.7
No	47 (100%)	236 (97%)	9 (100%)	1 (100%)	
Yes	0 (0%)	7 (2.9%)	0 (0%)	0 (0%)	

**Croup**					> 0.9
No	47 (100%)	242 (100%)	9 (100%)	1 (100%)	
Yes	0 (0%)	1 (0.4%)	0 (0%)	0 (0%)	

**Others**					**0.010**
No	34 (72%)	177 (73%)	2 (22%)	1 (100%)	
Yes	13 (28%)	66 (27%)	7 (78%)	0 (0%)	

*Note:* Bolded valuse in last column means statistical significance

**Table 8:** Patients with complaint of cough and fever at initial visit and revisit (*N* = 300).

	*n*	%
Cough	84	28%
Fever	115	38.3%

## 4. Discussion

This study investigated the etiology of pediatric ED RVs, focusing on initial and subsequent visit management and outcomes while correlating these factors with patient demographics at KASCH in Riyadh, Saudi Arabia. A substantial proportion of the heterogeneous cohort consisted of young children (0–3 years), which is consistent with other similar studies [14–16], with 27% presenting with chronic conditions. The prevalence of asthma among these children (12%) aligns with its recognition as a common chronic illness in pediatric populations [2].

Respiratory symptoms, particularly persistent cough and dyspnea, emerged as the primary drivers of ED utilization. Several factors, including weather fluctuations, seasonal influenza, and frequent exposure to viral pathogens in early childhood, may contribute to the elevated RV rates for respiratory conditions. This corroborates previous research linking respiratory illnesses to increased ED visits [5]. The most frequent reasons for RVs included persistent cough (34%), persistent fever (25%), and vomiting (20%). Notably, symptom persistence across visits was substantial: 28% of the cohort presented with cough at both the initial visit and the RV, and 38.3% reported fever at both time points (Table 8). This pattern of symptom nonresolution reinforces the self‐limiting yet protracted nature of URTIs in children and may reflect inadequate parental education regarding expected illness trajectories at the time of initial discharge. These findings underscore the ongoing challenge in managing self‐limiting but distressing symptoms in young children, particularly when parents are uncertain about clinical deterioration.

A notable finding warranting clinical attention is the antibiotic prescribing pattern observed across both visits. Antibiotics were prescribed in 28% of initial visits and 29% of RVs. Given that URTI accounted for 52% of index diagnoses—a condition predominantly of viral etiology in the pediatric age group—this prescribing rate raises concern for antibiotic overuse in the emergency setting. The persistence of antibiotic prescribing at the RV encounter, at a nearly identical rate to the index visit, suggests limited reassessment or de‐escalation between contacts and potentially reflects diagnostic uncertainty, parental expectation, or institutional prescribing culture. Antibiotic overprescribing in pediatric URTI is a well‐documented global challenge with implications for antimicrobial resistance and patient safety [2, 5]. These findings highlight an opportunity for targeted antimicrobial stewardship interventions in the pediatric ED, including structured decision‐support tools, clear return precaution guidance, and parental education regarding the self‐limiting nature of viral respiratory illness. Future studies with individual‐level linkage of initial and RV prescribing data would better characterize the true rate of antibiotic continuation versus new initiation.

The high discharge rate (98%) at initial visits is typical, as many acute pediatric illnesses can be managed in outpatient settings, aligning with Goldman et al.′s findings of predominantly discharged patients and a minority requiring admission [17]. However, the 16% admission rate upon RV, mirroring Seiler et al.′s observations [9], underscores the potential for disease progression [18, 19]. Alternatively, missed diagnoses during the initial assessment could contribute [1], a phenomenon also noted by DePiero et al., who documented 12 instances of initial diagnostic errors [19]. This emphasizes the importance of thorough assessments during the initial visit to reduce the likelihood of RVs due to unresolved or worsening conditions.

Notably, children aged 0–3 years exhibited a higher propensity for 72‐h ED RVs at KASCH, corroborating findings by Burokiene et al. and Goh et al., who also reported the highest RV rates in this age group [1, 5]. This increased vulnerability to RVs among the youngest children may be attributable to their frequent exposure to viral pathogens. Additionally, the nonspecific and often subtle presentation of illness in preverbal children can make it difficult for parents to assess severity, potentially leading to a lower threshold for returning to the ED [1, 20].

We observed sex‐based differences, with males showing higher admission rates, in contrast to Goldman et al.′s findings, which reported no correlation between admission rates and either sex or age [17]. This warrants further investigation to understand the differences and underlying mechanisms in our community.

The seasonal distribution of ED visits peaked in the fall, potentially linked to seasonal allergens and respiratory viruses, although this contrasts with some studies reporting a spring peak [1]. Both initial and RVs frequently occurred in the evening, possibly reflecting limited primary healthcare access during these hours, a finding consistent with existing literature [5, 21, 22]. This pattern highlights the need for accessible after‐hours care options to divert nonurgent ED visits.

Most patients initially presented and revisited via self‐referral, suggesting a gap in primary care utilization for managing common pediatric complaints. Strengthening primary care services and improving access to timely care could potentially reduce ED RVs for nonurgent conditions. The similar distribution of diagnoses at initial and RVs reinforces the importance of accurate initial diagnosis and targeted management of common pediatric illnesses.

This study has limitations, including its retrospective design, single‐center setting at one tertiary hospital in Riyadh, and the limited sample size collected over a relatively short period, which restricts the generalizability of the findings beyond comparable tertiary institutions. The 14‐month study period, in which March and April appear twice, may introduce a degree of seasonal imbalance that should be considered when interpreting seasonal data. Additionally, individual‐level linkage of antibiotic prescriptions across visits was not feasible in this retrospective dataset, limiting the precision of antibiotic continuity analysis. Our results highlight the need for broader, prospective, multicenter studies with larger sample sizes and longer follow‐up periods to generate findings applicable across the Saudi healthcare system and the wider region. Qualitative studies exploring parental perspectives on ED utilization and RV decisions could provide valuable insights for developing targeted interventions to reduce unnecessary RVs and improve pediatric care.

## 5. Conclusion

This study investigated the etiology of pediatric ED RVs and characterized management outcomes at KASCH, Riyadh, Saudi Arabia. Persistent respiratory symptoms, notably cough and dyspnea, were identified as the leading causes of RVs, with asthma being the most prevalent chronic disease. The predominance of URTI as both the initial and RV diagnosis, combined with antibiotic prescribing rates of 28%–29% across both encounters, underscores the importance of antimicrobial stewardship and parental education in the pediatric ED setting. Accurate initial diagnosis and appropriate management are crucial for reducing ED RV rates. Furthermore, patient age and sex influenced admission rates and RV likelihood. These findings offer institution‐specific insights that may inform quality improvement efforts at comparable tertiary pediatric facilities and underscore the need for future multicenter research to establish regionally applicable evidence.

## Funding

No funding was received for this manuscript.

## Ethics Statement

The research project was approved by the Institutional Review Board (IRB) of King Abdullah International Medical Research Center (KAIMRC), with IRB Approval Number IRB/1608/23 and IRB approval date of 06 July 2023, and conducted in accordance with the Declaration of Helsinki. Informed consent was waived, as this study was conducted as a retrospective chart review.

## Conflicts of Interest

The authors declare no conflicts of interest.

## Data Availability

All patient data were coded at the time of collection and deidentified prior to analysis. Patient confidentiality was strictly maintained throughout the study; data were used solely for research purposes and accessible only to authorized members of the research team, in compliance with institutional data protection policies.
